# Anopheline Larval Habitats Seasonality and Species Distribution: A Prerequisite for Effective Targeted Larval Habitats Control Programmes

**DOI:** 10.1371/journal.pone.0052084

**Published:** 2012-12-18

**Authors:** Eliningaya J. Kweka, Guofa Zhou, Stephen Munga, Ming-Chieh Lee, Harrysone E. Atieli, Mramba Nyindo, Andrew K. Githeko, Guiyun Yan

**Affiliations:** 1 Climate and Human Health Research Unit, Kenya Medical Research Institute, Kisumu, Kenya; 2 Kilimanjaro Christian Medical University College, Tumaini University Makumira, Moshi, Tanzania; 3 Program in Public Health, College of Health Sciences, University of California Irvine, Irvine, California, United States of America; University of South Alabama, United States of America

## Abstract

**Background:**

Larval control is of paramount importance in the reduction of malaria vector abundance and subsequent disease transmission reduction. Understanding larval habitat succession and its ecology in different land use managements and cropping systems can give an insight for effective larval source management practices. This study investigated larval habitat succession and ecological parameters which influence larval abundance in malaria epidemic prone areas of western Kenya.

**Methods and Findings:**

A total of 51 aquatic habitats positive for anopheline larvae were surveyed and visited once a week for a period of 85 weeks in succession. Habitats were selected and identified. Mosquito larval species, physico-chemical parameters, habitat size, grass cover, crop cycle and distance to nearest house were recorded. Polymerase chain reaction revealed that *An. gambiae* s.*l* was the most dominant vector species comprised of *An.gambiae* s.s (77.60%) and *An.arabiensis* (18.34%), the remaining 4.06% had no amplification by polymerase chain reaction. Physico-chemical parameters and habitat size significantly influenced abundance of *An. gambiae* s.s (*P* = 0.024) and *An. arabiensis* (*P* = 0.002) larvae. Further, larval species abundance was influenced by crop cycle (*P*≤0.001), grass cover (*P*≤0.001), while distance to nearest houses significantly influenced the abundance of mosquito species larvae (r = 0.920;*P*≤0.001). The number of predator species influenced mosquito larval abundance in different habitat types. Crop weeding significantly influenced with the abundance of *An.gambiae* s.l (*P*≤0.001) when preceded with fertilizer application. Significantly higher anopheline larval abundance was recorded in habitats in pasture compared to farmland (*P* = 0.002). When habitat stability and habitat types were considered, hoof print were the most productive followed by disused goldmines.

**Conclusion:**

These findings suggest that implementation of effective larval control programme should be targeted with larval habitats succession information when larval habitats are fewer and manageable. Crop cycles and distance from habitats to household should be considered as effective information in planning larval control.

## Introduction

In most African highlands the natural forest ecology has been changing in the recent past due to human population increase and demand for more agricultural land, thus favoring mosquito survivorship and parasite development [1], [2], [3]. This has resulted into continuous local transmission and increased risk of malaria epidemics in highlands [4], [5], [6], [7], [8]. Malaria transmission in highlands have been fostered with rise in temperature as the output of high rate of deforestation and land use changes [6], [9], [10], [11], [12], [13], [14]. The increased human population has put more pressure on land resources resulting into reclamation of swamps to increase food security and deforestation to create land for settlement and pasture for grazing livestock. This creates more habitats which are exposed to more sunlight which in turn increases water temperature and shortens the developmental cycle of immature stages of malaria vectors [7], [12], [13], [15], [16]. The rise in temperature has increased developmental rate of parasites in adult mosquitoes [1], [2]. The main malaria vectors in these highlands of western Kenya are *An. gambiae* s.s, *An. arabiensis* and *An. funestus* [7], [11], [17], [18], [19], [20].

Malaria control in this region relies heavily on use of insecticides treated bed nets (ITNs), Indoor residual spray (IRS) and diagnosis and treatment of all active malaria cases [21], [22], [23], [24]. In highlands of western Kenya, larval habitats are concentrated on the valley bottom due to regional topography [7], [9], [14], [25]. The abundance of aquatic stages of malaria vectors have been found to increase with cropping seasons in different parts of Africa [7], [9], [14], [26], [27], [28], [29]. Therefore a clear understanding of the *An. gambiae* s.*l* larvae succession in different land use types in relation to crop cycles can be of additional value in integrated vector control programs. Recent studies have shown that larval control has major potential impact on malaria cases and vectors [30]. For example, a study in western Kenya highland showed that, combination of habitat larviciding and ITNs use, provided better protection than use of ITNs alone [31].

Larval control has proved to be effective in different parts of malaria endemic regions when habitats are few and manageable [32], [33]. Our previous study in western Kenya highlands revealed that, different mosquito larval species prefer different habitats in both dry and wet seasons [7]. Understanding habitats succession and *An. gambiae* s.*l* larvae abundance is important in designing the possible vector aquatic stage control timing. Even though larval abundance may not be an effective method of predicting vector productivity in a certain habitats [7], [13], [19], it is still a good indicator of vector availability in the area [11]. Although several larval ecology studies have been conducted in western Kenya highlands, there is still limited information on larval habitat succession in relation to crop rotations in different land use types. This information however, is critical for planning an effective larval control program. For example, in Ethiopia it was demonstrated that crop cycles influence mosquito larval abundance [27], [28], [29]. Larval habitats in different land use types have been found to have high variations in larval abundance and productivity [7], [9], [12], [13], [14], [34]. In another study in western Kenya, habitat productivity measured by number of emerging adult mosquitoes per meter square was found not to be uniform between habitat types [7]. To verify if these results are a common phenomenon in all highlands, more studies were required under different habitat and land use types to assess if crop cycles, physico-chemical parameters, grass cover and nearness to house influence mosquito larval abundance.

Thus this study aimed at understanding i) abundance of aquatic stages of anopheline mosquitoes in different habitats and land use management ii) influence of habitat physico-chemical parameters and temperature on mosquito larval abundance, iii) habitat types and other factors such as predators and grass cover and their impact on larval abundance, and iv) influence of crop cycle on anopheline larval abundance. The information presented in this paper is important for strategic larval control in highlands of western Kenya.

## Materials and Methods

### Description of the Study Site

**Figure 1:**
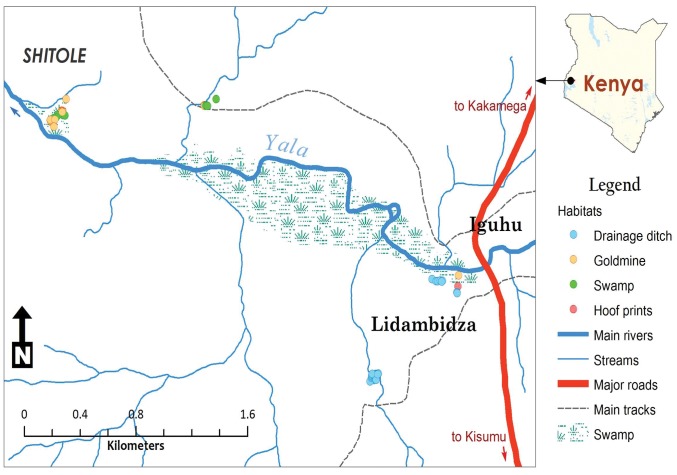
A map showing the study sites and habitat types used during the study period in western Kenya Highlands.

The study was conducted at Iguhu village within Ikolomani constituency, a malaria epidemic - prone area in western Kenya highlands (Figure 1). The study area has a population of approximately 104,669, according to Kenya census report in 2009. The main agricultural activities mostly cultivation of maize, vegetable, banana, beans in small scale farming. Tea is grown as as a cash crop on a small scale. Goats, cattle, sheep and poultry are owned by members of the community. The topography of the study area is valley and hills [9], [14], [34], [35]. The deforestation rate in the study area has been increasing due to increased growth in human population and demand of more land for cultivation and timber for house construction and this has brought about change in weather conditions [1], [2], [6]. Small scale mining in the study village has made remarkable increase in larval habitats.

### Habitat Selection and Study Site Mapping

**Figure 2:**
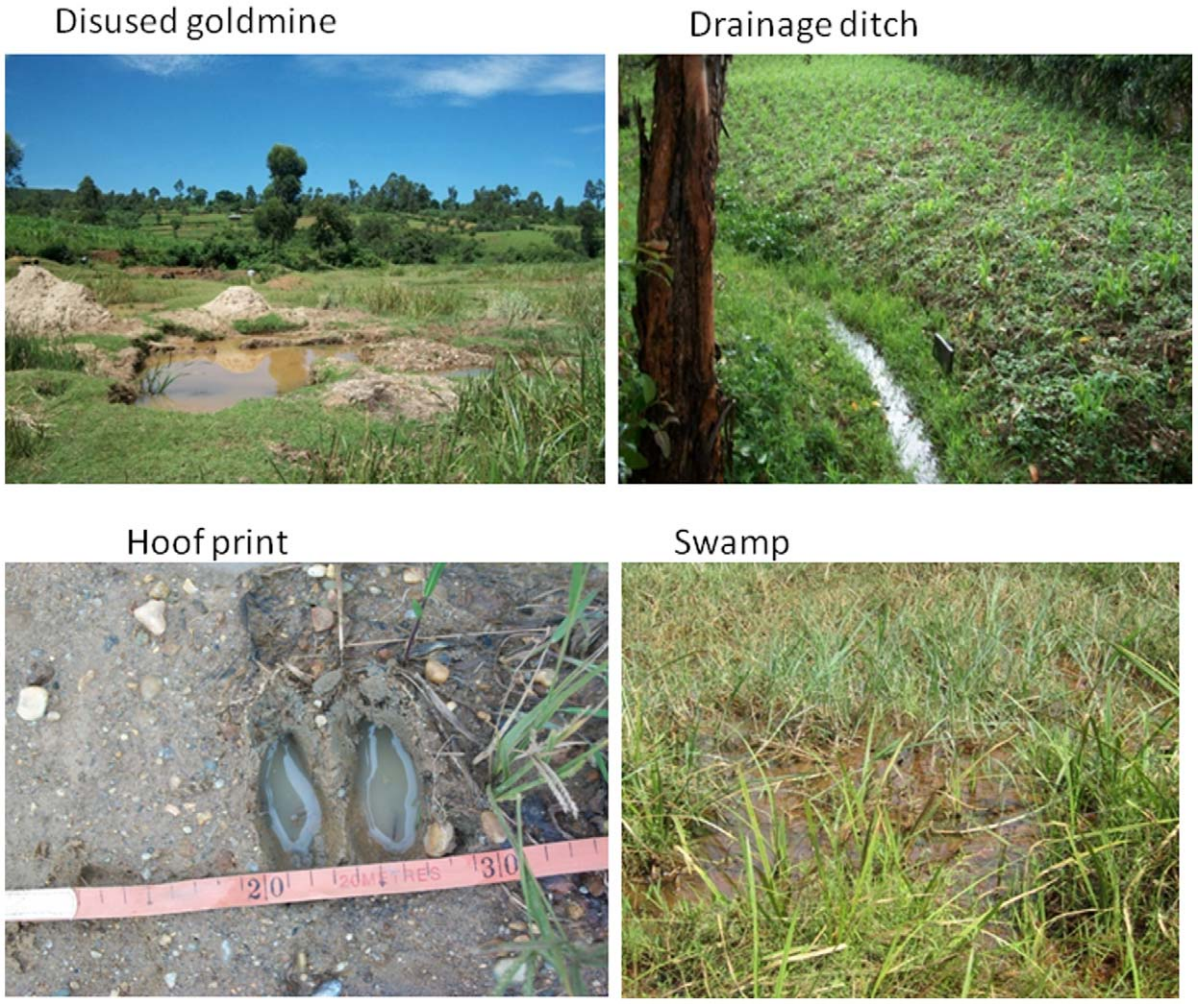
Types of habitats used for larvae abundance in different land use types.

Identification of larval habitats positive for aquatic stages of anophelines was conducted in June, 2009. First, a thorough search for anopheline larvae positive habitats was done in different land use types in which four habitat types were: swamp, drainage ditches, hoof print and disused goldmine (Figure 2). These land use types have been described in details elsewhere [7], [13]. A habitat was selected for inclusion in the study if it had anopheline larvae at the time it was first sampled. Each selected habitat was given a permanent identification number using a wooden mark and was geo-referenced using a handheld Garmin Global Positioning System unit (eTrex Venture HC)**.**

### Crop Cycles and Seasons

Plant development stages and land preparation were considered as crop cycles stages and grouped into three main categories: land preparation, crop weeding and crop flowering. Seasons were defined by absence or presence of rains of different intensities. There were three classes of seasons identified during the study period: rainy season, short rainy season and dry season.

### Larvae Sampling and Identification

Larval sampling was done using standard 350 mL dipper (BioQuip Products, Inc. California, USA) done once every week. Water collected by larval dippers was emptied into a white basin and checked for mosquito larvae. Larvae present were identified morphologically using keys developed by Gillies and Coetzee [36]. Number of larvae from different habitats with different water volume was calculated into number of larvae per ten dips and meanwhile the depth, length and width of the habitat were measure. Larvae were transported to laboratory for storage in absolute ethanol and subsequently in a refridgerator at 4°C till when they were being identified. Members of *An. gambiae* complex were further identified to species level using rDNA-polymerase chain reaction (PCR) protocol by Scott *et al.,* [37].

### Measurement of Physico-chemical Variables

Temperature, pH, Chlorophyll a, Nitrates, Nitrites and Phosphates content were measured in all habitats. Chlorophyll a was determined using the hand held machine, Aquafluor (Sunnyvale, CA, USA). pH was measured using portable machine (pH Tester 10, Oaklon, USA). Nitrates, Nitrites and Phosphates contents were measured using spectrophotometer technique by use of portable smart colorimeter (LaMOTTE Company, 002 Washington, Chestertown, MD 21620, USA). All the physico-chemical parameters were measured on site at the time of mosquito larval sampling.

### Statistical Analyses

Species diversity index equation was used for analysis of mosquito species abundance and richness. A diversity index is a mathematical measure of species diversity and abundance in a community. Diversity indices provide more information about community composition than simply species richness (i.e., the number of species present); they also take the relative abundances of different species into account, which is useful information in control design. Therefore, species diversity index(SDI) was computed using the Simpson’s Diversity Index equation [38], [39], for measuring the species heterogeneity or homogeneity for all 85 weeks in different land use and habitat types, as shown below:



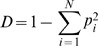
where *P_i_* is the fraction of a species which belong to the i-th species, that, 0≤ D ≤0 with values near zero corresponding to highly diverse or heterogeneous ecosystems and values near one corresponding to more homogeneous ecosystems. Where; *p* = proportion of individuals in each species and N = number of species.

Stepwise multiple regression analysis was used to compare the differences in larval abundance in land use types, grass covers, seasonality, crop cycles, and habitat-types. Also interactions of the factors such as crop cycles, seasons and land use types were included in analysis.

Impact of physico-chemical parameters (Nitrates, Nitrites Phosphates), chlorophyll a and temperature, habitat size and predator species abundance and distance to nearest house were tested using multivariate regression. Data analysis was performed using PASW statistics version 18 (SPSS Inc., Chicago, IL).

### Ethical Approval

Ethical approval for this study was granted by the Kenya Medical Research Institute, National Ethical Review Committee and University of California, Irvine ethical review board, under main project named “Ecology of African highland malaria (II), SSC No. 1382”. Before implementation of the study, village leaders and elders were called for a meeting to explain the essence of the study. The verbal consent to visit the habitats in selected sites (both in farmland and pasture) was obtained from all land owners in all selected sites.

## Results

### Larval Species Abundance and Diversity

**Figure 3:**
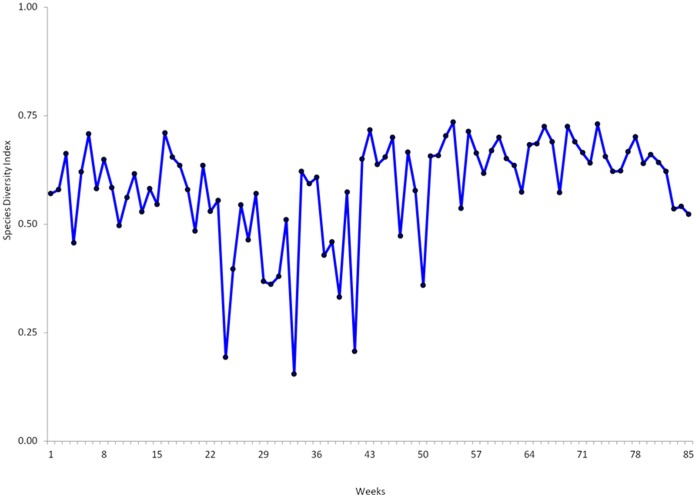
Mosquitoes larvae diversity index by Simpson model in 85 weeks of survey.

**Figure 4:**
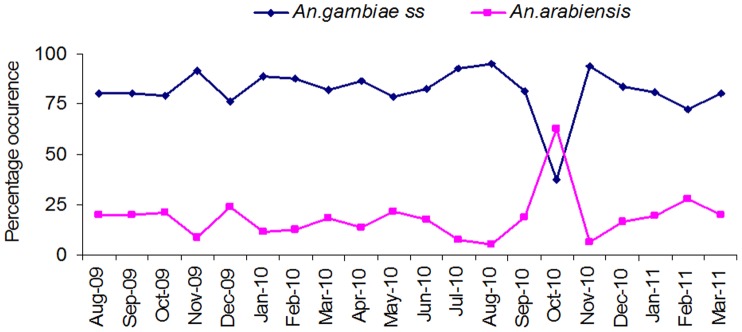
*Anopheles gambiae* s.s and *An. arabiensis* larvae abundance and dynamics among identified specimen throughout 85 weeks of field surveys.

Larval habitats positive for anopheline larvae were mainly found in farm and pasture land use types, with no positive habitat found in forest. A total of 46,846 immature stages of mosquitoes were sampled from all the habitats during the study period. Out of these, *An. gambiae* s.*l* accounted for 48.21% (n = 22583), *An. funestus* larvae and pupae accounted for 11.59% (n = 5428), other anopheline including *An. coustani, An. squamous*; *An. ziemanni* and *An. implexus* accounted for 9.46% (n = 4433) and culicine larvae accounted for 30.74% (n = 14402). Due to time and resource limitations only 616 *An. gambiae* s.*l* larvae were identified using PCR from random samples selection at different weeks of study duration. Among 616 specimens of *An. gambiae* s.*l* identified with PCR method, 77.60% (n = 478) were identified as *An. gambiae s.s* while 18.34% (n = 113) were *An. arabiensis* and specimens with no PCR product amplifications were constituted4.06% (n = 25). Over the study period, the Simpson model showed that, there were variations in species diversity over the sampling weeks (Figure 3) and in habitat types, with disused goldmines having a Diversity Index (DI)of 0.69, hoof prints (DI = 0.64), swamps (DI = 0.62) and drainage ditches (DI = 0.66). When the data was analyzed according to land use types, the index did not show any variations in the species diversity, farmland (DI = 0.66) and pasture (DI = 0.66). Overall, larval densities as measured by larvae per dip were: drainage ditches (3.84 larvae/dip), hoof prints (4.95 larvae/dip), disused goldmines (4.43 larvae/dip) and swamps (4.39 larvae/dip) which was statistically insignificant. *An. gambiae s.s* and *An. arabiensis* larvae abundance among identified specimens varied significantly throughout the study period (Figure 4).

### Crop Cycles and Larvae Abundance

**Figure 5:**
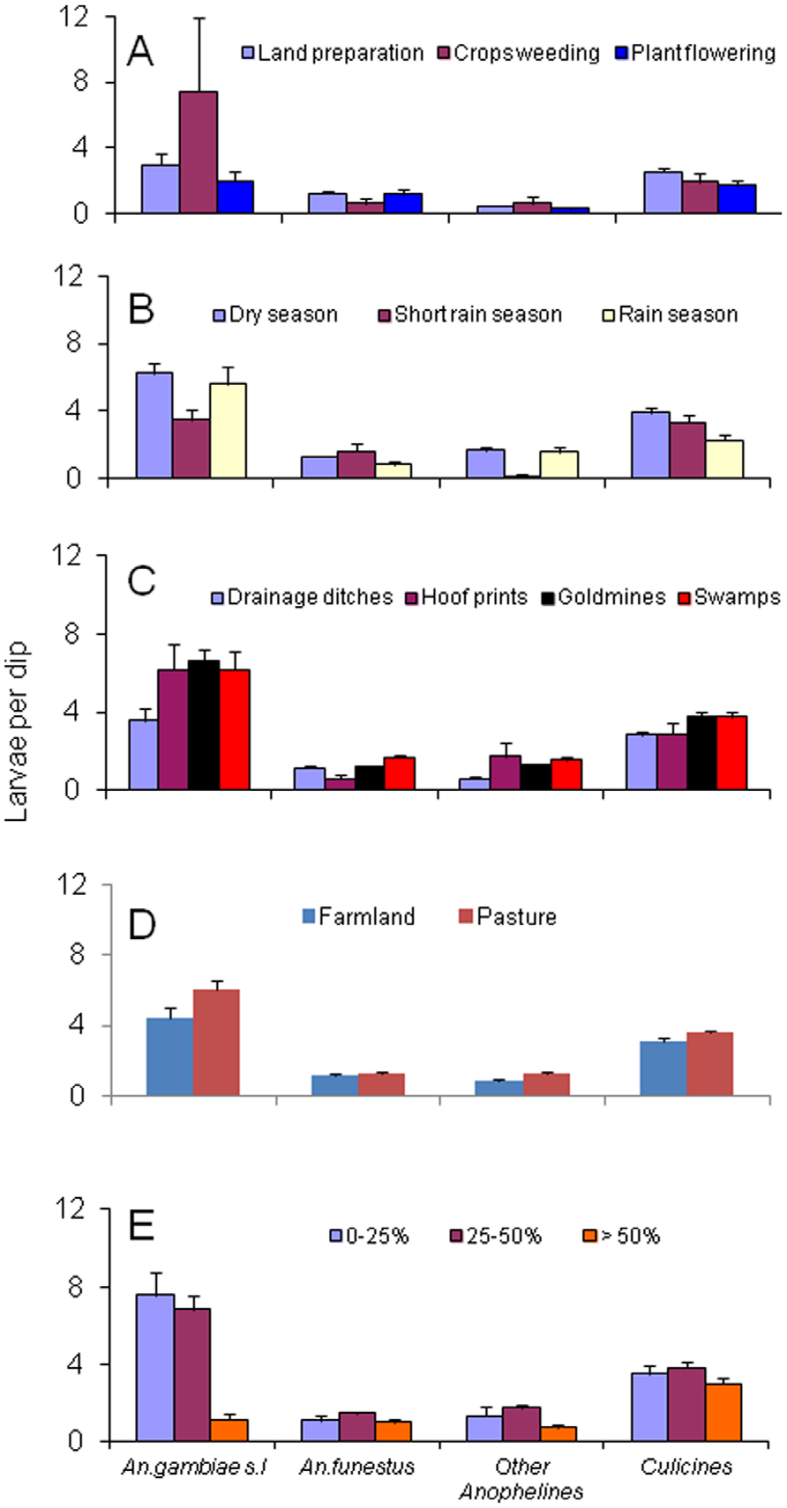
Mosquitoes larvae per dip in different (5A) crop cycle, (5B) seasonality, (5C) habitat types and (5D) land use types during 85 weeks of larvae abundance survey.

Larval abundance varied significantly between all the three cropping cycles (land preparation, crops weeding and plant flowering): *An. gambiae* s.l (F = 8.14, DF = 2, *P*≤0.001), other Anopheline (*P*≤0.001) and Culicines (*P*≤0.001). On the other hand, *An. funestus* larval abundance was statistically insignificant (*P* = 0.277) between the cropping cycles (Figure 5A).

### Seasonality and Larval Abundance

In all three seasons (rainy, short rain and dry seasons), *An. gambiae s.l* were found to have no significant differences among seasons (F = 2.14, DF = 2, *P* = 0.119), while *An. funestus* (*P* = 0.046), other Anopheline (*P* = 0.003) and Culicines (*P* = 0.007) were significantly different among the cropping cycle (Figure 5B).

### Habitats Types, Landuse Types and Larval Abundance

Mosquito larval abundance was significantly higher among the habitat types; *An. gambiae* s.*l* (F = 2.80, DF = 3, *P = *0.004), *An.funestus* (*P*≤0.001), other anopheline (*P*≤0.001) and culicine (*P*≤0.001) (Figure 5C). Overall, *An. gambiae s.l* larval abundance was significantly higher in pasture (F = 4.229, df = 1, *P = *0.040), other Anophelines (F = 13.436, df = 1, *P*≤0.001) and Culicines species (F = 4.519, df = 1, *P* = 0.034) than in farmland. In contrast, *An. funestus* larval abundance was statistically insignificant (F = 0.826, df = 1, *P = *0.363) between the land use types (Figure 5D )**.** When all anopheline larvae were pooled together, pastures had significantly higher larval abundance than farmland (F = 10.052, df = 1, *P = *0.002).

### Grass Cover and Larval Abundance

**Figure 6:**
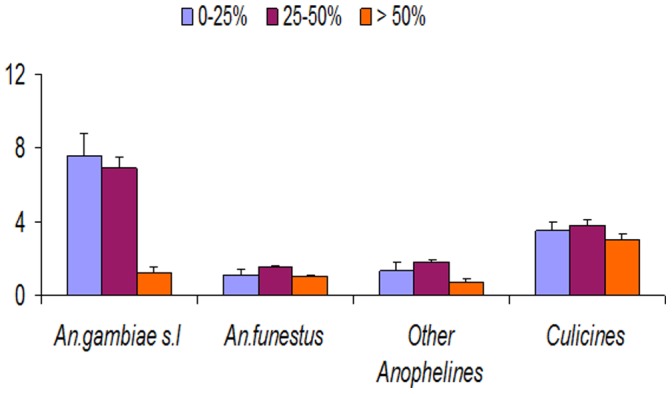
Mosquito larvae per dip in different grass cover (canopy) during 85 weeks of larvae abundance survey.

Grass cover significantly influenced the presence and abundance of mosquito larvae: *An. gambiae* s.l (F = 15.182, df = 2, *P*≤0.001) and other anopheles species (F = 3.401, df = 2, *P = *0.034). Increased grass cover over the habitats reduced mosquito larval abundance. However grass cover had no influence on mosquito larval abundance (Figure 6):*An. funestus* (F = 2.695, df = 2, *P = *0.069) and culicine species (F = 1.582, df = 2, *P* = 0.207). When all mosquito larvae were pooled together it was found that, increase in grass cover significantly reduced larval abundance(F = 15.247, df = 2, *P*≤0.001).

### Physico-chemical Parameters and Larvae Abundance

In larval habitats succession experiments *An.gambiae s.l* larval abundance was significantly influenced by elevated levels of Nitrates and pH (Table 1). Other physical and chemical habitat parameters had no significant influence on *An.gambiae s.l* larval abundance (Table 1). *An. funestus*, other anophelines and culicine species larval abundance had no significant association with the habitats’ physico-chemical characteristics (Table 1)**.**

**Table 1:** Multiple regression analysis for assessment of mosquito larvae abundance in presence of different physico-chemical parameters in habitats (significant values are bolded).

	*An. gambiae* s.l		*An. funestus*		Other Anopheline		Culicine larvae	
Parameters	t	*P*-value	t	*P*-value	t	*P*-value	t	*P*-value
Nitrates (ppm)	2.141	**<0.034**	−1.178	0.240	−1.822	0.070	−1.960	0.094
Nitrites (ppm)	4.177	**<0.001**	0.437	0.662	−0.300	0.764	1.687	0.052
pH	0.224	0.823	0.226	0.822	−0.846	0.399	−1.463	0.145
Phosphates (ppm)	1.309	0.192	−0.539	0.591	−0.056	0.118	−1.118	0.265
Chlorophyll a	2.199	**<0.001**	−0.011	0.591	0.012	0.574	0.026	0.201
Temperature (max)	0.145	0.885	−0.685	0.494	1.911	0.058	1.371	0.172
Temperature (min)	−0.435	0.664	−0.522	0.602	−0.769	0.443	−2.057	**0.041**

### Predator Species and Larval Abundance

Among the predator species, *Gambusia affinis,* backswimmer and dragon fly nymphs significant influence on *An. gambiae s.l*, *An. funestus*, other anopheline and Culicines larval abundance (Table 2). Belestoma presence significantly reduced the larval abundance of *An. funestus*. Presence of tadpoles in the habitats was associated with increased culicine larval abundance (Table 2).

**Table 2:** Multiple regression analysis for mosquito larvae abundance influenced the presence of the five predator species (significant values are bolded).

	*An. gambiae* s.l		*An. funestus*		Other Anopheline		Culicine larvae	
	t	*P*-value	t	*P*-value	t	*P*-value	t	*P*-value
Tadpoles	0.946	0.346	0.566	0.572	1.040	0.175	0.470	0.639
*Gambusia affinis*	2.050	**<0.001**	1.549	0.975	0.975	**<0.001**	0.087	0.931
Backswimmer	2.350	**0.020**	1.727	0.086	2.740	**0.007**	1.532	0.127
Dragon fly nymph	1.038	**0.012**	1.329	0.186	1.505	0.134	0.207	0.836
Belestoma	1.359	0.176	0.898	0.371	0.452	0.652	0.459	0.647

### Distance of Habitat to Houses

The distance from habitat to houses was found to be influence *An. gambiae* s.l larvae abundance (r = 0.920, *P*<0.001). However abundance of other mosquito larvae was not influenced by the distance to nearest house (*An. funestus* (r = 0.026, *P* = 0.355), other anopheline (r = 0.002, *P = *0.878*)* and culicines (r = 0.022, *P* = 0.142).

### Habitat Size and Larvae Abundance

Habitat size significantly influenced larval abundance of *An. gambiae s.l*, *An. funestus,* other anopheline and culicines. (Table 3). Significantly higher numbers of *An. gambiae s.l* larval abundance occurred in small size habitats while *An. funestus* larvae were associated with larger water bodies. The other Anopheline and culicine species had a preference for medium sized water bodies (Table 3).

**Table 3:** Multiple regression analysis to investigate mosquito larvae abundance in different habitat size, habitat types and number of dips made (significant values are bolded).

	*An.gambiae s.l*		*An.funestus*		Other Anopheline		Culicine larvae	
	t	*P*-value	t	*P*-value	t	*P*-value	t	*P*-value
Habitat size	0.020	0.189	5.333	**<0.001**	2.966	**<0.001**	3.999	**<0.001**
Number of dips	4.299	**<0.001**	3.983	**<0.001**	4.555	**<0.001**	4.113	**<0.001**
Habitat type	2.798	**<0.001**	0.023	0.136	0.023	0.125	−0.002	0.878

## Discussion

The findings of the current study have revealed that, pasture land use types have higher abundance of mosquitoe larvae than in farmland. Moderately higher occurrence of Nitrates and pH concentrations were found to increase abundance of *An.gambiae s.l* larvae. Grass cover was found to cause a decrease of *An. gambiae s.l* abundance and increase *An. funestus* and other anopheline larvae. Currently, there is growing interest in investing on mosquito larval control and the feasibility of reducing malaria vector populations through environmental management which has been under investigation in different ecological settings in African malaria endemic countries [16], [30], [32], [40], [41], [42].

This study has demonstrated dominance of *An. gambiae s.l* over other species for 85 weeks of the larval habitats follow up in both pasture and farmland uses. This was indicated by the Simpson diversity index model which showed species homogeneity dominance in many weeks close to 1 and species heterogeneity in 78 weeks out of 85 weeks of follow up (Figure 3). Mosquito larval abundance was significantly higher in pastures than in farmlands with dominance of *An.gambiae s.l*. These findings are similar to previous studies that demonstrated more larvae in pastures where habitats are more exposed to sunlight for a long time. providing suitable habitats for larval growth and oviposition by gravid mosquitoes [7], [10], [12], [13], [43], [44], [45], [46]. These habitats which are exposed to sunlit have warmer temperatures thus accelerating the development of the aquatic stages of mosquitoes and consequently may reduce the chances of higher mortality and predation risks [47]. In all larval habitats surveyed, larval abundance was relatively species dependant, *An. funestus* and culicine species colonized large water bodies (swamps and large open disused goldmines) and this data is similar to previous findings [7], [10], [13], [15], [36], [46], [48]. *An. gambiae s.l* high abundance was significantly higher in small open disused goldmines, hoof prints and in cultivated swamps, which is similar to ecological phenomenon explained by other researchers findings [7], [10], [13], [15], [36], [46], [48].

*An. gambiae s.l* larval abundance in farmland was found to dominate during different cropping cycles (crop cycles were categorized on three stages depending on crop stage and land preparation; these stages were land preparation (land tilling), crop weeding and crop flowering) during the crop weeding cycle which was preceded with application of fertilizer. during crops growing This is contrary to what other studies found in farmlands that flowering plant cycle has higher abundance of *An.gambiae s.l* in the habitats [26], [27], [28], [29]. The findings of this study suggest that, the food sources in larval habitats in farmlands are more complex and larvae might have other sources of food apart of flowering maize pollen. Hence, the prediction of the food sources for mosquito larvae in this study could not be ascertained by cropping cycles alone. In ecological studies, larval habitats have been found to have the complex food web which supports the larvae of different species at a time [26], [27], [28], [29].

It is interesting to note that, higher concentrations of physico-chemical parameters, i.e. Nitrate and pH in larval habitats had significant influence abundance of *An.gambiae s.l* larvae. Previous studies, have shown similar results of physico-chemical parameters influencing anopheline larval abundance [19], [49], [50]. In contrast, one study in western Kenya however did not show any significant influence between nitrite, phosphate and *An.gambiae s.l* larval abundance [19]. Application of nitrogenous fertilizers in different agro-ecosystem has been demonstrated to lower water turbidity and consequently significantly influence mosquito larval abundance [49], [50], [51], [52], [53] since anopheline mosquitoes prefer to oviposit in areas with lower turbidity [54], [55]. It is likely that, temporary, shallow and open small aquatic habitats attract more gravid *An. gambiae* s.*s* and *An. arabiensis* by visual cues to oviposit in habitats probably due to visibility of habitat substrate and absence of predators [36], [48], [56]. However, mosquitoes differ in their preference to the type, size, turbidity, algal cover, and stability of the habitat [44], [48], [56], [57], factors which determine larval density [57], [58], [59]. This study found that larval abundance in habitats varied with rains seasonality. Dry and short rainy seasons had the highest larval abundance in habitats for all species. Results of the current study are similar to other studies in western Kenya which studied habitat productivity estimation [7], [10], [11], [13], [19], [60], [61] and found that the larval abundance and adult productivity decreased in long rainy reason while in short rainy and early dry seasons they increase. The lower larval density in early short rain and in long rainy seasons might be attributed and explained by unexpected flush effect (washing of eggs and larvae from habitats) which was similar to findings from other studies in western Kenya [7], [62]. These results suggest that during dry season and at the end of short rainy season are the most suitable time for effective targeted larval control programmes as habitats are highly productive, fewer and manageable. This might be the case due to the restricted habitats for oviposition during dry season and habitat stability during the short rains when they are not flooded and washed away [7], [10], [35], [61]. This would give an opportunity to utilize the seasonality of habitats and larval abundance for the effective suppression of larvae and adult mosquito abundance when larval habitats are fewer and confined in a manageable places. Fillinger and others found that, the effective suppression of larval habitats and subsequently adult density reduction can be attained during semi and dry seasons [30], [32], [41]. In our study, at the end of dry season (October, 2010, Figure 4) the abundance of *An. gambiae s.s*. larvae decreased while that of *An. arabiensis* increased in the dry season and is likely a disease transmission risk to human population in absence of cattle to provide blood meal for *An. arabiensis* **.** In these scenarios, larval populations are likely regulated by hydro-period [63] and/or controphic? and intraspecific exploitative competition [54].

The distance of larval habitat to houses in this study had positive influence with *An. gambiae s.s* larvae abundance which shows the risk of increased indoor vector density. Other studies have demonstrated that, since *An. gambiae s.l* is closely associated with human dwellings. Therefore, it will utilize the closest habitats for oviposition [15], [64], [65], [66]. Gravid *An. gambiae* **s.s** utilizes the habitats within close proximity to the homesteads for oviposition as an evolutionary strategy for energy conservation. This finding meant that habitats far away from the households had lower *An. gambiae s.s* larval densities due to few numbers of gravid females of *An. gambiae s.s*. using them for oviposition.

Other Anopheline species (*An. squamous, An. coustani, An. ziemanni* ) larvae were less abundant in habitats close to human dwellings. *Anopheles funestus* has a relatively long aquatic stage (>21 days in the lowlands) and this requires stable habitats such as large water bodies with emergent grass that may not be close to human dwellings.

Predators’ significantly influenced mosquito larval abundance, for example; the presence of *G. affinis* and dragon fly nymph in habitats had great impact on larvae reduction of all mosquito larvae. Belestoma and backswimmers had great impact on *An. gambiae* and culicines larvae reduction. On the other hand, presence of tadpoles had no impact on larval reduction hence mosquito larvae increased in habitats where tadpoles dominated. This is contrary to what was found in laboratory and semi- field experiments [67], [68]. The predation effect on the different larval species has shown similar trends to other studies conducted using the same five predators [67]. Predators such as *G. affinis*, belestoma, backswimmer and dragon fly nymph are potential predators for *An. gambiae s.l* larvae and could be evaluated in small scale in natural ecological settings as biological control agents. In habitats with larval predators, top-down and bottom-up processes are likely to be important as joint determinants of community structure [54], [67], [69]. Mosquito oviposition might have been restricted in habitats with predators which give danger sensing cue signals to endanger the survivorship of the mosquitoes off springs [68], [69], [70].

*An. gambiae s.l* larval abundance decreased with the increase of grass cover height while *An. funestus* and other anopheline species increased with grass cover abundance Culicine larval abundance was similar in habitats with both short and high grass cover size. Larval abundance and mosquitoes productivity studies in different ecological settings have found similar species responses with grass cover increase [16], [42], [51]. Ecological studies have shown that, higher grass cover reduces sunlight penetration to the habitat which affect the algae biomass photosynthesis efficiency and other aquatic forms which are other sources of food to mosquito larvae [71], [72]. Grass cover influences oviposition site selection by mosquitoes hence directly effect on larvae abundance as observed by other researchers [61], [71], [73], [74]. Understanding larval habitats ecology and larval habitats succession in western Kenya highlands is of importance in designing an effective targeted malaria vector habitats control program in these highlands.

### Conclusion

The findings of this study suggest that, implementation of effective larval control should be targeted when larval habitats are fewer and manageable with high larvae abundance during dry and end of short rain seasons. Disused goldmines, swamps and drainage ditches should be targeted during dry season while hoof prints should be considered in short rain season.
